# Improving Metabolic Health in Obese Male Mice via Diet and Exercise Restores Embryo Development and Fetal Growth

**DOI:** 10.1371/journal.pone.0071459

**Published:** 2013-08-19

**Authors:** Nicole O. McPherson, Hassan W. Bakos, Julie A. Owens, Brian P. Setchell, Michelle Lane

**Affiliations:** 1 School of Paediatrics and Reproductive Health, Discipline of Obstetrics and Gynaecology, University of Adelaide, South Australia, Australia; 2 School of Medicine, Discipline of Medicine, University of Adelaide, South Australia, Australia; 3 School of Medical Sciences, Discipline of Anatomy, University of Adelaide, South Australia, Australia; 4 Repromed, Dulwich, South Australia, Australia; State University of Rio de Janeiro, Biomedical Center, Institute of Biology, Brazil

## Abstract

Paternal obesity is now clearly associated with or causal of impaired embryo and fetal development and reduced pregnancy rates in humans and rodents. This appears to be a result of reduced blastocyst potential. Whether these adverse embryo and fetal outcomes can be ameliorated by interventions to reduce paternal obesity has not been established. Here, male mice fed a high fat diet (HFD) to induce obesity were used, to determine if early embryo and fetal development is improved by interventions of diet (CD) and/or exercise to reduce adiposity and improve metabolism. Exercise and to a lesser extent CD in obese males improved embryo development rates, with increased cell to cell contacts in the compacting embryo measured by E-cadherin in exercise interventions and subsequently, increased blastocyst trophectoderm (TE), inner cell mass (ICM) and epiblast cell numbers. Implantation rates and fetal development from resulting blastocysts were also improved by exercise in obese males. Additionally, all interventions to obese males increased fetal weight, with CD alone and exercise alone, also increasing fetal crown-rump length. Measures of embryo and fetal development correlated with paternal measures of glycaemia, insulin action and serum lipids regardless of paternal adiposity or intervention, suggesting a link between paternal metabolic health and subsequent embryo and fetal development. This is the first study to show that improvements to metabolic health of obese males through diet and exercise can improve embryo and fetal development, suggesting such interventions are likely to improve offspring health.

## Introduction

Worldwide obesity is epidemic, with 200 million men and 300 million women over the age of 20 currently classified as obese [1]. While, maternal obesity is well established to adversely affect the oocyte and negatively impact the establishment of pregnancy [2]–[4], there is now mounting evidence that paternal obesity is also implicated in gamete health and pregnancy outcomes [5]–[7]. An overweight or obese male with a female partner of normal body mass index (BMI), has an increased odds ratio for a longer time to conceive, compared with couples where both are of normal weight [8], [9]. Studies of couples undergoing assisted reproductive technology (ART) have established that male obesity is associated with reduced pregnancy rates and increased pregnancy loss [5]–[7]. This phenomenon seems to be as a result of reduced sperm binding and fertilisation rates, as well as impaired blastocyst development [5], [10], [11].

Similarly, in experimental rodent models of paternal obesity where males are fed a high fat diet (HFD) to induce obesity with or without impaired glucose control, perturbed sperm function, with reduced sperm motility, increased oxidative stress and DNA damage are seen [12]–[14]. When these obese males were then mated to normal weight females, they exhibited impaired embryo development, and reduced implantation and live birth rates [11], [15], [16].

Recently it has been established that exercise and caloric restriction in obese males can reduce adiposity and improve sexual function. One study in humans examined 43 obese men who were placed on a 14 week diet and exercise weight loss program and demonstrated improved total sperm count and morphology in those men who lost the greatest weight [17]. Gastric bypass surgery and weight loss in obese men have shown similar results with improvements to sex hormone profiles and sexual function [18]. Additionally, diet and exercise interventions in a mouse model of male obesity induced by a HFD has recently shown that sperm function was highly correlated with their metabolic health, with normalisation of serum glucose, cholesterol, triglycerides, free fatty acids (FFA) and insulin, associated with restoration of sperm motility, morphology, oxidative stress, DNA damage and sperm binding [13].

Thus weight loss strategies have shown promise in restoring sperm quality of obese males in both rodents and humans, but no studies to date have determined whether weight loss and improved metabolic state in obese males can reverse their associated impaired embryo health and pregnancy outcomes. We have therefore induced obesity in male mice with additional diet and/or exercise interventions, previously reported to reduce adiposity, improve metabolic state and sperm function [13] and determined if early embryo, fetal development and pregnancy rate can be restored. We hypothesise, that weight loss and or an improvement to metabolic health via diet and exercise interventions in obese males will improve subsequent embryo and fetal health similar to levels of normal weight males.

## Methods

### Ethics Statement

This study was carried out in strict accordance with the Australian code of practice for the care and use of animals for scientific purposes. The use and care of all animals used in the study was approved by the Animal Ethics Committee of The University of Adelaide.

### Animals and Diet

Five week old male C57BL6 mice (n = 40) were randomly assigned to one of two diets for an initial period of 9 weeks: 1) control diet (CD) (SF04-057; Specialty Feeds, Perth, Australia, (Table S1); or 2) a high fat diet (HFD) high in fat and nutrient matched (SF00-219; Specialty Feeds, Perth, Australia, Table S1). Diets used in the study have been previously shown to increase adiposity in male mice, without affecting glucose tolerance after 9 weeks [15], [19]–[21]. After the initial feeding period, males allocated to the HFD were further allocated to one of the following interventions for a further period of 9 weeks: 1) continuation of a HFD (HH) (n = 8); 2) change to a CD (HC) (n = 8); 3) continuation of a HFD with exercise (HE) (n = 8); 4) change to a CD with exercise (HCE) (n = 8). Mice allocated to the CD during the initial feeding period were also fed a CD during the intervention period as a baseline control (CC) (n = 7). These interventions have previously been shown to reduce adiposity in those obese males that undergo diet interventions and to improve metabolic parameters in those obese males that undergo exercise and or diet interventions [13]. Animals were individually housed for the entire study and fed ad lib.

### Exercise Intervention (Swimming)

The swimming exercise regime was followed as previously described [13]. Briefly male mice were placed into tank containing warm water at a constant temperature of 32°C±1°C to swim freely for the set time period. For the first 2 weeks mice swam for 3×15 min periods over 7 days with one days rest in between swimming sessions [22]. This allowed time for the mice to become accustomed to exercise regime and the swimming tank. For the remainder of the intervention (6 weeks) mice swam for 3×30 min training sessions each week with at least one days rest in between swimming sessions to simulate light exercise. This imposes light exercise in comparison with a moderate training program in which mice freely swam for 60 min, 5 days a week, for 18 weeks [23].

### Body Composition

Individual body weights of the males were recorded weekly during the pre and post intervention periods. At pre intervention week 9 (14 weeks of age) and at the end of intervention week 9 (23 weeks of age) whole body composition of lean mass, bone density, adiposity were measured by a dual-emission X-ray absorptiometry machine (DEXA) (Piximus, Ge Lunar, Wisconsin, USA) as previously described [13]. Additionally, selected organs (liver, kidneys and pancreas) and reproductive tissues (gonadal fat, testes, seminal vesicles) were collected and weighed at the end of the intervention period (intervention week 9, 23 weeks of age) after males were humanely killed by cervical dislocation post cardiac puncture.

### Serum Metabolite Analysis

Blood was sampled from the tail vein after six hours fasting, at the end of pre intervention week 9 (14 weeks of age) and post intervention week 9 (23 weeks of age), and blood glucose was measured by a glucometer (Hemocue, Angelholm, Sweden). At the end of the intervention period (intervention week 9, 23 weeks of age), a fasted blood sample was obtained via cardiac puncture under anaesthetic with 2% Isoflurane (1-chloro-2,2,2-trifluoroethyldifluoromethylether) (Veterinary Companies of Australia, Kings Park, Australia) (44 hr post swimming training for exercise intervention mice) for measurement of serum cholesterol, FFAs, triglycerides and leptin. Serum cholesterol, FFAs and triglycerides were measured by enzymatic analysis on a Hitachi 912 automated sample system, as previously described [13] and serum leptin levels were determined using a mouse leptin ELISA kit (Cat# 90030) as per the manufacturer's instructions (Crystal Chem Inc, Downer Grove, USA).

### Intraperitoneal Glucose Tolerance Test (GTT) and Insulin Tolerance Test (ITT)

A GTT and ITT was performed at the beginning of intervention week 6 and 7 respectively (20 and 21 weeks of age respectively) after 6 h of fasting by intra-peritoneal (IP) injection of 2 g/kg of 25% D-glucose solution for GTT or during a fed state by IP injection of 0.75 IU of human insulin (Actapid®, Novo Nordisk, Bagsvaerd, Denmark) for a ITT. GTT and ITT were performed on rest days for exercise intervention mice (HE and HCE). Glucose concentrations were measured using a glucometer (Hemocue, Angelholm, Sweden) at time points 0 (pre-bolus basal), 15, 30, 60 and 120 min on whole blood collected from the tail vein through a tail cut. Data were expressed as mean blood glucose concentration per group as area under curve (AUC) for glucose and area above the curve (AAC) for insulin.

### Embryo Collection

3–4 week old C57BL6 female mice who were fed standard chow (Table S1) were given an intraperitoneal (IP) injection of 5 IU Pregnant Mare's Serum Gonadotropin (PMSG) (Folligon, Invervet, Bendigo, Australia), followed 48 h later with an IP injection of 5 IU human Chorionic Gonadotropin (hCG) (Pregnyl, Organon, Sydney, Australia) to induce ovulation as per [24], [25]. Following the hCG injection female mice were individually placed with a C57BL6 male between intervention weeks 7–9 (21–23 weeks of age). Each male (n = 8 HH, HC, HE, HCE and n = 7 CC) had the opportunity to mate with 6 super ovulated females at independent times. Successful mating was assessed the following morning by the presence of a vaginal plug. There was no significant difference between mating rates between groups. Successful mating occurred in 6 HH and HCE males and 7 CC, HC and HE males. Each individual male produced between 30 to 80 zygotes from 2–4 females (Table S2). Males that under went swimming exercise (HE and HCE) were mated on alternate days to exercise and each male from all treatment groups had a least 1 days rest in between mating. At 22–24 h post hCG female mice were humanly killed by cervical dislocation and, cumulus enclosed zygotes were collected and placed in MOPS at 37°C and denuded of cumulus cells by 1 min incubation with 0.5 mg/ml hyaluronidase. Zygotes were washed twice in MOPS and once in G1 medium before culture in G1 media [26].

### Embryo Culture

Embryos were cultured in groups of 10 in 20 µl drops of G1 under paraffin oil (Merek, New Jersey, USA) for 48 h at 37°C in 5% O_2,_ 6% CO_2_ and 89% N_2_. Embryos were then washed and cultured in medium G2 for a further 48 h to the blastocyst stage. On-time embryo development was assessed at 43 h post hCG (day 2, cleavage), 67 h post hCG (day 3, compaction), 98 h post hCG (day 4, early blastocyst) and 115 h post hCG (day 5, late blastocyst). All embryo culture dishes were prepared 4 h prior to embryo culture to allow for gassing and temperature equilibration with 24 replicate experiments performed.

### E-cadherin in Embryos

**Figure 1:**
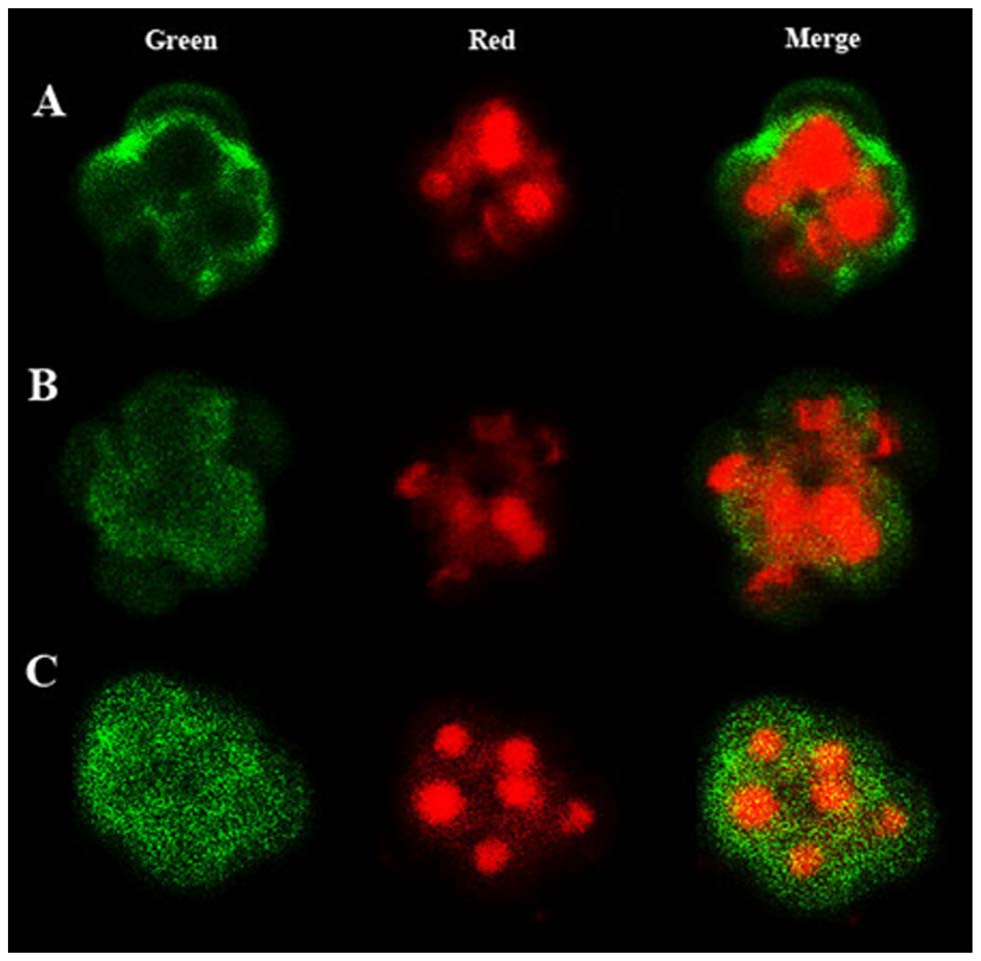
E-cadherin Staining Patterns. **A** E-cadherin localisation to cell to cell contacts. **B** E-cadherin localisation to both cell to cell contacts and cytoplasm. **C** E-cadherin localisation to the cytoplasm. Green images represent E-cadherin staining, red images represent nuclear staining and merge images represent a compression of both. Pictures are representative of a single z-section through embryos.

**Figure 2:**
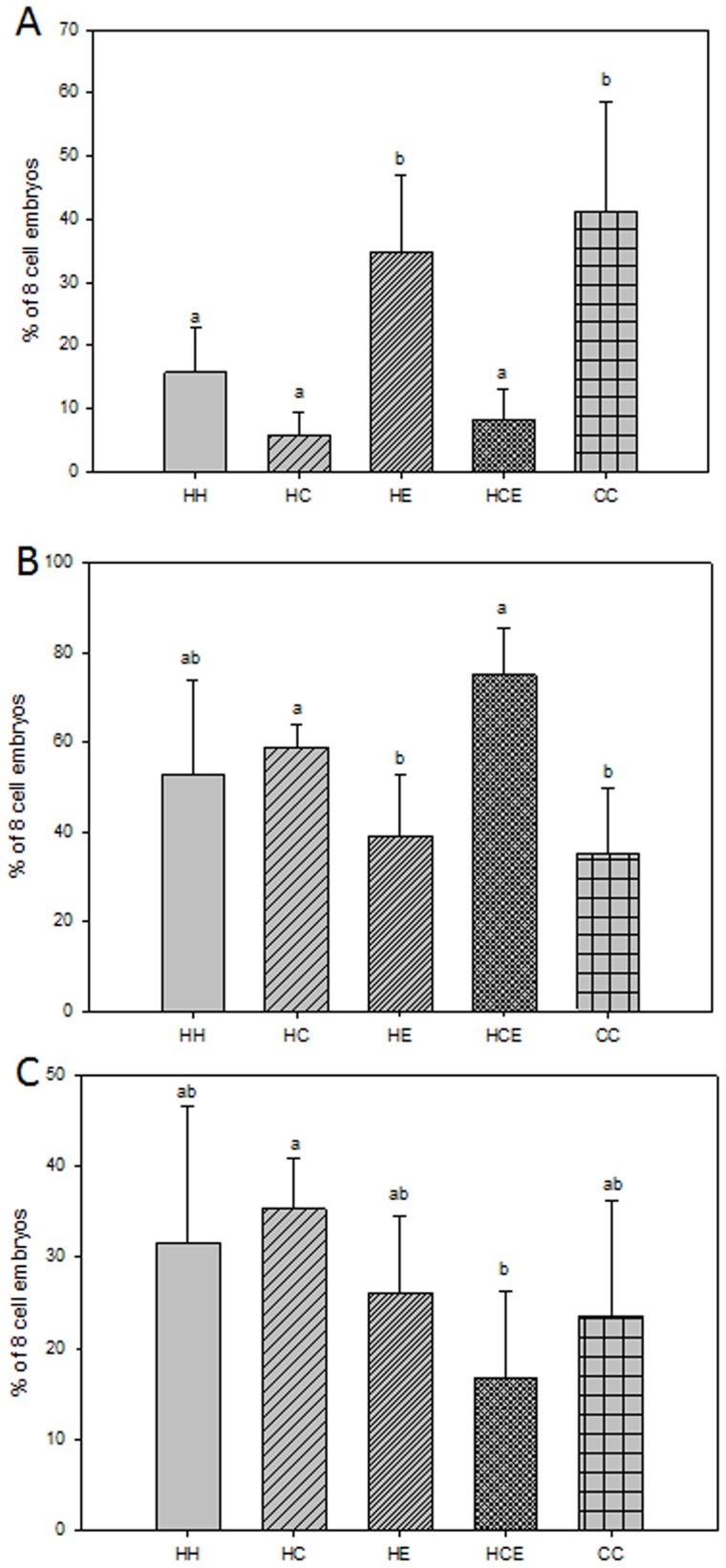
The Effect of Diet and Exercise of Obese Males on E-cadherin Staining Patterns in Compacting Embryos. **A** The percentage of 8 cell embryos with E-cadherin localised to cell to cell contacts. **B** The percentage of 8 cell embryos with E-cadherin localised to both cell to cell contacts and cytoplasm. **C** The percentage of 8 cell embryos with E-cadherin localised to the cytoplasm. Date represents 3 HH, HCE and 4 CC, HC and HE males which is representative of 3–7 embryos per father per group.

The localisation of E-cadherin in embryos was determined by a modified immunofluorescence protocol as previously described [27]. Briefly, 8-cell embryos were collected at 67 h post hCG, fixed in 4% paraformaldehyde overnight and stored in 0.1 M glycine. Embryos were permeabilised in 0.25% TritonX-100 (PBS-TX) for 45 min and placed into blocking solution (1∶10 donkey serum) and primary antibody (1∶200, Rabbit anti E-cadherin, Abcam, Cambridge, UK; ab53033) at 4°C overnight. The following day embryos were washed through PBS-TX and placed into secondary antibody (1∶200, Donkey anti Rabbit Alex 488, Life Technologies, Invitrogen, Mulgrave, Australia; A-21206) at 37°C for 2 h. Following another wash in PBS-TX embryos were placed in 0.25 mg/ml of propidium iodine (PI) for 5 min to stain the nucleus. For a negative control the primary antibody was omitted from the reaction. Embryos were loaded in glycerol and allowed to settle to the bottom before been imaged blinded by the same individual using confocal microscopy. E-cadherin localisation were determined by characterising if 8-cell embryos had either 1 of 3 staining patterns observed during confocal z-sectioning; 1) E-cadherin at cell to cell contacts (Fig. 1A), 2) E-cadherin at both cell to cell contacts and cytoplasm (Fig. 1B) and 3) E-cadherin only in the cytoplasm (Fig. 1C). The numbers of embryos for each staining pattern for each treatment group were expressed as a percentage.

### Blastocyst DNA Damage

The number of apoptotic cells in each blastocyst were determined at day 5 of embryo development (115 h post hCG) using TUNEL (Terminal deoxynucleotidyl Transferase Biotin-dUTP Nick End Labelling) (In Situ Cell Death detection kit, Roche Molecular Biochemicals, Indianapolis, IN) as described previously [28]. The numbers of apoptotic cells were expressed as a percentage of total cell number for each blastocyst. A positive control was used where blastocyst were incubated in the presence of DNase I (Invitrogen, Mulgrave, Australia) and a negative control where blastocysts were incubated only in the fluorescent buffer.

### Assessment of ICM, Trophectoderm and Epiblast Cell Number (Nanog and Oct4 Staining)

To determine the number of epiblast cells within the ICM, day 5 blastocysts were further grown in the above culturing system for an additional 24 hours (Day 6, 139 h post hCG) to allows time for epiblast cells to be detected in the ICM by Nanog and Oct4 antibodies and stained using our previously published method [29]. Briefly blastocysts were fixed in 4% paraformaldehyde overnight at 4°C and neutralised in 0.1 M glycine. Blastocysts were permeabilised in PBS-TX for 15 min at room temperature (RT) and blocked overnight at 4°C in 10% normal donkey serum (Sigma, D9663). Blastocysts were then incubated in primary antibodies rabbit anti Nanog (Cozmo Bio, Tokyo, Japan; REC-RCAB0002P) (1∶200) and goat anti Oct4 (Santa Cruz, California, USA; SC-8628) (1∶100) for 1½ h at 37°C. Following washing in PBS-TX blastocysts were incubated in secondary antibodies donkey anti rabbit Alexa 488 (Life Technologies, Invitrogen, Mulgrave, Australia; A-21206) (1∶100) and donkey anti goat Alexa 594 (Life Technologies, Invitrogen, Mulgrave, Australia; A-11058) (1∶100) for 2 h at RT. To further determine cell nuclei blastocyst were incubated in DAPI for 2–3 min at RT before being loaded in glycerol and counted using fluorescent microscopy. Total cell number (TCN) was assessed by nuclei stained DAPI; inner cell mass (ICM) cell number nuclei also stained positive for Oct4, and epiblast cell number nuclei additionally stained positive for Nanog. Trophectoderm cell number (TE) was determined by subtracting ICM from TCN. For a negative control the primary antibodies were omitted from the reaction.

### Embryo Transfer

Morphological similar expanded or hatched blastocysts (115 h post HCG) were transferred into day 4 pseudo pregnant 10 week old Swiss female mice (−1 day asynchronous). Embryos from each treatment were randomly allocated to each uterine horn with each mother having six blastocysts from 2 different treatment groups (transferred to each uterine horn). Twelve to eighteen embryos were transferred into 2–3 mothers per father from 5 HE and HCE and 6 CC, HH and HC males. Mothers were maintained on standard chow (Table S1) until day 18 of pregnancy. On day 18 of pregnancy, mothers were humanely killed by cervical dislocation and implantations, numbers of fetuses and resorptions were determined per uterine horn for calculation of implantation rates, percentage of fetuses and percentage of fetuses per total number of implantations. Fetuses and placentas were dissected and removed of connective tissue, umbilical cords and maternal arterial space (apostrophe) for placentas. Crown rump length and weights of resultant fetuses and placentas were measured.

### Statistical Analysis

All data were expressed as mean ± SEM and checked for normality using a Kolmogorov-Smirnov test and equal variance using a Levene's test. All statistical analysis was performed in SPSS (SPSS Version 18, SPSS Inc., Chicago, USA). A p value <0.05 was considered to be significant.

***Weight gain data, body composition and DEXA body composition*** in our founder males were analysed by a one way ANOVA with an LSD post hoc test.

***Embryo cell development, embryo DNA damage and embryo cell number*** were expressed per father and analysed by a one way ANOVA with a LSD post hoc test between groups. Nanog staining was analysed by Mann-Whitney U test between groups as the data are not normally distributed.

***Fetal weight and size, placental weight and size and fetal to placental weight ratios*** there was no interaction between litter size and fetal weights and therefore fetuses and placentas were expressed per father and analysed by a univariate generalized linear model with a LSD post hoc test between groups. Cohort of founders was fitted as a covariate.

***Implantation rates and E-cadherin staining patterns*** were analysed by a Chi Square between groups.

***Correlations between founder's serum metabolites and embryo and fetal health*** were determined by a Pearson's Rho test and expressed as the correlation coefficient (CCO) along with its corresponding p value and number of observations. To determine if paternal adiposity was contributing to the correlations of serum metabolites to embryo and fetal health additional partial correlations controlling for paternal adiposity were also included.

## Results

### Effect of Diet and Exercise on Embryo Development

There was no change in cleavage rates to the 2-cell stage between any of the treatment groups (p>0.05, Table 1). Males fed a HH produced a decreased number of embryos at the 8-cell/compacting stage on day 3 compared with CC (p<0.05, Table 1), with combined diet and exercise intervention (HCE) advancing development to the 8 cell/compacting stage compared with HH and HC (p<0.05, Table 1). Diet alone (HC) or exercise alone (HE) did not alter development to the 8-cell/compacting stage, compared with HH (p>0.05, Table 1). This advancement in embryo development from CC males was also evident on day 4 with reduced numbers of early blastocysts contributing to total blastocyst numbers compared with HH males (p<0.05, Table 1). All interventions (HC, HE and HCE) displayed advanced blastocyst development on day 4 with increasing numbers of hatching blastocysts compared with HH (p<0.05, Table 1) and a decrease in expanded blastocysts compared with CC (p<0.05, Table 1). By day 5 of embryo development exercise interventions (HE and HCE) maintained their increased numbers of hatching blastocysts compared with HH (p<0.05, Table 1). There was no difference between total blastocysts, expanded blastocysts or hatching blastocysts between the remaining treatment groups (HC, CC and HH) indicating a slower rate of development in embryos from HH males, rather than a failure to develop.

**Table 1:** The Effect of Diet and Exercise of Obese Males on Embryo Development.

Diet/ Intervention	CC	HH	HC	HE	HCE
***Day 2***					
Cleavage	88.6±5.3	88.9±4.7	89.2±2.5	90.3±3.7	94.3±6.2
***Day 3***					
8 cell/ compacting	71.4±5.2^a^	54.2. ±5.5^b^	58.2± 5.4^b^	64.3±4.9^ab^	72.0±5.6^a^
Compacting	14.7±3.7^ab^	15.7±3.9^ab^	9.6±3.7^a^	8.4±3.5^a^	22.2±4.0^b^
***Day 4***					
Total blastocyst	75.5±5.0	74.1±5.3	74.7±5.1	82.2±4.7	86.2±5.4
Early blastocyst	10.3±3.6^a^	20.7±4.1^b^	13.8± 3.8^ab^	16.1±3.0^ab^	12.2±3.5^ab^
Expanded blastocyst	27.3±3.1^a^	21.3±3.2^ab^	14.0± 3.1^b^	13.9±2.9^b^	14.4±3.3^b^
Hatching blastocyst	45.3±5.0^ab^	34.3±5.3^b^	47.9±5.1^ac^	53.0±4.7^ac^	59.2±5.4^c^
***Day 5***					
Total blastocyst	86.4±4.5	83.1±4.7	83.2±4.5	84.1±4.2	87.5±4.8
Expanded blastocyst	19.6±3.6	21.1±3.8	16.5±3.7	10.9±3.4	12.3±3.9
Hatching blastocyst	65.2±5.0^ab^	55.4±5.3^a^	61.9± 4.9^ab^	72.4±4.7^b^	74.5±5.6^b^

Embryo development is expressed as percentage of embryos ± SEM at each stage of development per father. Data represents 6 HH and HCE males and 7 CC, HC and HCE males which is representative of at least 300 embryos per treatment group (for exact breakdown of embryos per father see Table S2). On day 4 total blastocysts is made up of early, expanded and hatching blastocysts, while on day 5 of on time embryo development total blastocyst is made up of a combination of expanded and hatching blastocysts. Different letters denote significance at p<0.05.

### Effect of Diet and Exercise on Blastocyst Cell Numbers and DNA Damage

Blastocysts produced by males from the HH group had lower total cell numbers on day 5, compared with those of CC males (p<0.05, Table 2). All interventions (HC, HE and HCE) increased total blastocyst cell number on day 5, compared with HH (p<0.05, Table 2), restoring this to be comparable with the CC group (p>0.05, Table 2). This reduction in cell number from the HH was not as a result from increased apoptosis as there was no difference in the number or percentage of TUNEL positive cells in the blastocyst across groups (p>0.05, Table 2).

**Table 2:** The Effect of Diet and Exercise of Obese Males on Blastocyst Development.

Diet/Intervention	CC	HH	HC	HE	HCE
***Day 5***					
Total cell number	76.7±3.8^a^	64.3±3.6^b^	75.3±4.2^a^	74.9±3.5^a^	86.6±5.7^a^
TUNEL positive cells	3.73±1.10	4.54±1.00	3.50±0.87	3.76±0.87	3.59±0.50
DNA damage (%)	4.86±1.38	7.03±1.60	4.65±1.15	5.01±1.12	4.15±0.51
***Day 6***					
Total cell number (TCN)	98.3±6.3^a^	84.0±7.6^b^	100.0±3.7^a^	96.3±3.5^a^	99.7±6.2^a^
Trophectoderm cell number	83.4±5.5^a^	72.1±7.3^b^	86.6±1.3^a^	83.2±3.7^a^	85.6±5.3^a^
ICM cell number	15.0±1.29^a^	12.0±1.04^b^	12.9±1.05^ab^	13.6±0.53^ab^	14.2±1.04^a^
Epiblast cell number	7.2±0.71^a^	5.7±0.80^b^	6.2±0.73^ab^	6.2±0.45^ab^	6.6±0.84^ab^
Trophectoderm (% of TCN)	84.7±0.96	85.4±1.48	86.6±0.78	85.6±0.95	85.5±0.64
ICM (% of TCN)	15.3±0.96	14.8±1.41	13.4±1.39	14.4±0.95	14.5±0.64
Epiblast (% of ICM)	46.2±4.50	46.7±5.99	47.1±3.63	46.6±3.90	45.7±3.37

Data is expressed as mean ± SEM per father. Data represents 6 HH and HCE males and 7 CC, HC and HCE males which is representative of 5–8 embryos per father per group. Different letters denote significance at p<0.05.

### Effect of Diet and Exercise on Epiblast, ICM and Trophectoderm Cell Number

Embryos were further cultured for an additionally 24 hrs (day 6) to allow time for epiblast cells to be detected in the ICM by Nanog and Oct4 antibodies. All interventions (HC, HE and HCE) and CC had increased cell numbers (TCN and TE) compared with HH, similar to that observed on day 5 (p<0.05, Table 2). ICM and epiblast cell numbers were increased in blastocysts from the CC males compared with HH (p<0.05, Table 2) with combined diet and exercise interventions (HCE) also having an increase ICM cell numbers compared with HH (p<0.05, Table 2). Diet alone (HC) and exercise alone (HE) resulted in ICM and epiblast cell number not different to CC (p>0.05, Table 2). There was no change in the proportion of cells or percentage of cells allocated to the trophectoderm, ICM or epiblast between any of the treatment groups (p>0.05, Table 2).

### Effect of Diet and Exercise on Embryo Cell to Cell Contact

To further evaluate the effect of intervention treatments on rates of compaction after embryonic genome activation (after 2-cell stage) and therefore activation of the paternal contribution to the embryo, the localisation of E-cadherin (a marker of cell to cell contact) and one of the first paternally translated proteins within the developing 8-cell embryo [30], was assessed. CC and HE males had more 8-cell embryos with E-cadherin located at cell to cell contacts, compared with the other treatment groups (HH, HC and HCE, p<0.05, Fig. 2A). HCE males had more 8-cell embryos with E-cadherin located to both cell to cell contacts and cytoplasm compared with CC and HE males (p<0.05, Fig. 2B), but similar numbers compared with HH or HC males (p>0.05, Fig. 2B). HC males had more 8-cell embryos with E-cadherin located to the cytoplasm compared with HCE males (p<0.05, Fig. 2C). There was no change to the proportion of 8-cell embryos with E-cadherin located to the cytoplasm between the remaining treatment groups (HH, HC, HE, p>0.05, Fig. 2C).

### Effect of Diet and Exercise on Implantation and Fetal Viability

Blastocyst viability was assessed by transferring embryos to recipient mothers. Blastocysts produced from HH had reduced implantation rates compared with blastocysts from CC (p<0.05, Table 3). The combined diet and exercise intervention (HCE) was the only intervention to improve implantation rates compared with HH (p = 0.08, Table 3) with diet alone (HC) and exercise alone (HE) showing no improvements. There was no difference in fetal development, as assessed by the number of viable fetuses per the total number of blastocyst transferred, between any of the treatment groups (p>0.05, Table 3). Exercise intervention alone (HE) increased the percentage of implantations that resulted in viable fetuses, compared with CC and diet intervention alone (HC) (p<0.05, Table 3), with the remaining groups (HH and HCE) showing no change. HH had decreased fetal weights, lengths and placental weight compared with CC (p<0.05, Table 3). Any form of diet and/or exercise intervention (HC, HE, HCE) increased fetal weights compared with HH (HE and HCE p<0.05 and HC p = 0.08, Table 3); with diet alone (HC) and exercise alone (HE) additionally increasing crown-rump length compared with HH (p<0.05, Table 3). There was no change in placental weights between treatment groups (HC, HE and HCE) and HH (p>0.05, Table 3), however exercise alone did decrease placental weights compared with CC (p = 0.08, Table 3). There was no change in fetal to placenta weights ratio between any of the groups (p>0.05, Table 3).

**Table 3:** The Effect of Diet and Exercise of Obese Males on Subsequent Implantation and Fetal Development.

Diet/Intervention	CC	HH	HC	HE	HCE
Implantation (%)	98.5^a^	83.3^b^	85.9^bc^	84.7^bc^	93.1^aĉ^
Fetal (%)	15.3	23.6	14.4	26.4	16.0
Fetal/ Implantation (%)	14.5^a^	24.2^ab^	14.6^a^	27.7^b^	15.9^ab^
Fetal weight (mg)	773±31^a^	701±28^b^	776±42^â^	816±38^a^	818±31^a^
Crown-Rump length (mm)	18.4±0.4^a^	17.4±0.3^b^	18.6±0.5^a^	18.5±0.3^a^	18.0±0.4^ab^
Placental weight (mg)	113±3^a^	102±6^b^	109±8^ab^	104±5^b*^	102±10^ab^
Fetal: placenta ratio	6.8±0.5	7.0±0.5	7.3±0.3	7.7±0.3	7.4±0.2

Data is expressed as mean ± SEM per father. Implantation and fetal development data represents 5 HE and HCE and 6 CC, HH and HC males which is representative of 12–18 embryos transferred per father for each group. Fetal and placenta data represents 5 HE and HCE and 6 CC, HH and HC males which is representative of at least 10 fetuses per treatment group (for exact breakdown of number of fetuses per father see Table S2). Different letters denote significance at p<0.05. ?different to HH at p = 0.08 and *different to CC at p = 0.08.

Implantation (%) refers to the percentage of blastocyst that implanted into the uterus resultant from total number of embryos transferred determine by fetal numbers and resorptions. Fetal (%) refers to the percentage of embryos that formed fetus resultant for the total number of embryos transferred. Fetal/implantation (%) refers to the percentage of fetuses that formed from the total number of successful implantations.

### Associations between Paternal Metabolic and Hormonal state and Embryo and Fetal Growth and Development

Diet interventions (HC and HCE) reduced founder male body weight (HC −2.37±0.79 g and HCE −3.19±0.75 g), levels of adiposity (p<0.01), serum cholesterol (p<0.05), serum leptin (p<0.05), increased lean mass (p<0.05) and improved glucose tolerance (p<0.05) but maintained insulin resistance as measured by AAC compared with HH males (Table S3 and Table S4). Those receiving exercise intervention alone (HE) maintained their pre intervention body weight (−0.28±0.75 g) and level of adiposity (Table S3) with serum cholesterol and insulin resistance remaining comparable to HH males (p>0.05, Table S4) however, had a significant reduction in serum leptin levels (p<0.05, Table S4). Interventions including exercise (HE and HCE) reduced fasting serum glucose (HE p<0.05 and HCE p = 0.08) with an increased clearance of glucose after a GTT compared with HH males (p<0.05, Table S4).

Associations with metabolic and hormonal measures in founder males with embryo and fetal outcomes were further examined to identify any potentially influential factors due to the differences in embryo and fetal health observed in the intervention combinations. Paternal serum cholesterol correlated negatively with blastocyst cell number on day 5 (CCO −0.17, p = 0.01, n = 191) and day 6 embryos (CCO −0.52, p<0.01, n = 216), as well as TE cell number (CCO −0.54, p<0.01, n = 216), but positively with the percentage of DNA damaged cells in blastocysts (CCO 0.42, p = 0.01, n = 191). Paternal serum FFA correlated negatively with fetal to placental weight ratio (CCO −0.30, p = 0.01, n = 64) and percentage of ICM cells (CCO −0.50, p = 0.01, n = 210), but positively with the ratio of epiblast cells to ICM cells (CCO 0.51, p<0.01, n = 209) and the percentage of TE cells (CCO 0.51, p<0.01, n = 216). Paternal serum triglyceride correlated negatively with both total cell number (CCO −0.44, p<0.01, n = 216) and TE cell numbers (CCO −0.41, p<0.01, n = 216). Paternal insulin sensitivity (as measured by AAC during ITT) correlated negatively with the percentage of TE cells (CCO −0.44, p<0.01, n = 216) and percentage of DNA damaged cells in blastocysts (CCO −0.20, p<0.01, n = 191), but positively with the percentage of ICM cells (CCO 0.43, p<0.01, n = 210). Paternal fasting serum glucose correlated negatively with fetal to placental weight ratio (CCO −0.36, p<0.01, n = 64) and positively with placental weight (CCO 0.21, p = 0.05, n = 64). Additionally paternal adiposity correlated negatively with both TCN (CCO −0.51, p<0.01, n = 216) and TE cell number (CCO −0.49, p<0.0, n = 216) and positively with the percentage of DNA damaged cells in blastocysts (CCO 0.31, p = 0.03, n = 191).

When controlled for paternal adiposity the majority of the above correlations still held, indicating that paternal lipid status, glycaemia and insulin sensitivity are correlated to embryo and fetal health independently of paternal adiposity (Table S5).

## Discussion

There is emerging evidence that paternal obesity and related metabolic changes are associated with reduced embryo development and pregnancy establishment in both humans and rodent models [5]–[7], [15], [16], [31]. Here we show for the first time, using a well characterised model of diet induced obesity in the mouse, that diet and exercise interventions in the obese father, which either reduces adiposity or improves their metabolic state at the time of conception, improves subsequent embryo, pregnancy and fetal health. Previous studies of interventions to reverse obesity and metabolic syndrome in males had focused on influences on restoration of hormone profiles, sperm function and sexual dysfunction [17], [18], [32]–[36], but none had examined effects on embryo development and pregnancy establishment. Using diet induced obesity in male mice; we showed that the negative impact of paternal obesity on blastocyst development, blastocyst cell numbers, as well as subsequent implantation rate and fetal growth can be reversed through improvements to paternal metabolic health via diet and/or exercise. Further, the metabolic status of the father, including lipid status, glycaemia and insulin sensitivity are strongly associated with markers of blastocyst and fetal development or growth, independently of paternal adiposity or exposure to dietary or exercise interventions.

It has previously been shown and confirmed by this study that males fed a HFD produce embryos that have delayed development, with longer times to 1^st^, 2^nd^ and 3^rd^ cleavage and cavitation [11], [31] with a reduction in the numbers of on time developing embryos at the compacting, early blastocyst and late blastocyst stage [15]. Here we show for the first time that improvements to adiposity and metabolism via diet and/or exercise improve embryo development rates and blastocyst cell numbers in previous obese male mice. Specifically, the improved embryo development seen by those mice that underwent interventions involving exercise (HE and HCE) was evident after activation of the embryonic genome (in the mouse at the 2-cell stage); with more embryos reaching the 8 cell/compacting stage on day 3 and similarly for blastocysts on day 4 and day 5. In humans it has been shown that embryos that have successful implantation develop to the 8 cell stage faster than those embryos that couldn't implant [37] suggestive of improved implantation capability of embryos produced from exercise males.

Furthermore, we also confirmed the reductions in blastocyst cell numbers, including reduced numbers of ICM and TE cells, which have been observed from males fed a HFD [11], [15]. All diet and exercise interventions in obese male mice, increased blastocyst total cell numbers on both day 5 and day 6 of in vitro embryo development with improvements to both TE and to a lesser extent ICM cells. The late blastocyst is made up of two main cell types: 1; trophectoderm cells, which are necessary for embryo implantation and invasion and the establishment of the placenta; and 2; ICM cells, which can be split into epiblast cells that form the fetus and the primitive endoderm, that forms the extra embryonic tissue [38]. Changes to the numbers of cells or the distribution of the cells within the blastocyst can adversely affect the developing fetus, for an example, a reduction in numbers of ICM cells in a maternal diabetic rat model associated with fetal growth restriction [39]. Additionally in rodents blastocyst cell numbers as well as the proportion of cells within the late blastocysts have also been shown to positively correlate with implantation and pregnancy rates [26]. This suggests that exercise and diet intervention in obese males could potentially improve implantation and live birth rates, by restoring cell allocation and mitosis in the blastocyst.

In a clinical ART setting, obese males are associated with an increased rate of miscarriage, which may be as a result of sac only pregnancies [5] implying that cells within the embryo that form the fetus (i.e. epiblast cells) are impaired or missing. Here we directly show for the first time that diet induced obesity reduces the number of epiblast cells within the developing embryo and could potentially provide an explanation for the increased miscarriage rate seen in association with obese men [5]–[7]. Although diet and/or exercise interventions did not significantly improve epiblast cells number compared with HFD (HH) or CD (CC) they were still slightly increased compared to HFD mice, again suggesting likely improvements to implantation and pregnancy establishment.

Previous studies have also shown that blastocyst apoptosis is another marker of embryo health, with increasing apoptosis associated with reduced embryo survival, implantation rates and increases in early miscarriage [40]–[43]. Interestingly, in the current study, no differences were observed in relation to the levels of blastocyst apoptosis between the diet and exercise interventions or between HFD (HH) and CD (CC). This is contradictory to those findings found in Mitchell et al [15], where male mice fed a HFD for a period of 8 weeks showed increased rates of apoptosis in the late blastocysts. The differences in outcomes with the current study could be strain (C57BL6 compared with C57BL6xCBAF1) and age specific (23 weeks of age compared with 13 weeks) or related to the increased length of exposure to both the CD and HFD diets in the current study versus the previous study (18 weeks compared with 8 weeks).

The current study showed for the first time that diet induced obesity in resulted in changed localisation patterns of E-cadherin in compacting embryos, with more 8 cells having E-cadherin located in the cytoplasm from males fed a HFD, indicating a potential delay in establishment of the junctional communication between the blastomeres. However this potential delay was improved by exercise interventions with increased in E-cadherin observed in cell to cell contacts from these groups. The process of compaction in the developing embryo, involves polarisation of the cells, cell flattening and junctional communication [44]. E-cadherin is required in the developing embryo for the above processes, to allow compaction and cell division whilst also coordinating the cellular allocation and spatial segregation of the ICM and trophectoderm, with E-cadherin mutants unable to form a trophectoderm [30], [45], [46]. During embryo polarisation E-cadherin moves from the cytoplasm and becomes restricted to cell to cell contacts where it is stabilised before embryo compaction [47]. It therefore appears that E-cadherin in embryos localised in the cytoplasm compared with cell to cell contacts, is indicative of a delay in this polarisation and compaction. Changes to the localisation and levels of E-cadherin in compacting embryos where males were treated with cyclophosphamide [27], resulted in half of all compacting embryos with little or no cell contacts, with the immune reactivity of E-cadherin reduced and a significant delay in embryo development [27]. The improvements seen in those males that underwent exercise interventions suggests an advancement in development of compaction which may be contributing to the improvements in development and cell numbers seen in embryos derived from these males.

Previous studies in humans undergoing assisted reproductive technologies suggest that male obesity reduces implantation and therefore live birth rates [5]–[7]. Consistent with results observed in a previous rodent study of male obesity and embryo health [11], [15], the current study confirmed that males fed a high fat diet for the entire study had reduced implantation rates assessed at day 18 of gestation after embryo transfer, compared with those fed a CD. Interestingly, only the combined diet and exercise intervention (HCE) group improved implantation rates compared with males fed a HFD for the entire study. However it should be mentioned that morphological similar blastocysts from each group were used in embryo transfers which can predict implantation rates [37] and suggests that evaluation of a combined approach to weight loss in a clinical setting to evaluate the impact on implantation and therefore live birth rates is warranted.

Fetal development and size are not only determinants of a healthy pregnancy but can also pre determine the likelihood of developing adult chronic disease [48], [49]. Paternal exposure to high levels of x-rays as well as fathers' occupation is associated with small for gestational age babies independently of other confounding factors [50], [51], showing that paternal health at the time of conception can influence fetal health outcomes. Consistent with previous studies in rodents [11], [52] our HFD fed mice (HH) produced smaller fetuses compared with those fed a CD (CC), which has been linked with impaired metabolic and reproductive health in subsequent offspring [52], . Interestingly, all interventions (HC, HE, HCE) increased fetal weights compared to males fed a HFD (HH) with diet alone (HC) and exercise alone (HE) additionally increasing fetal lengths. This suggests that offspring born from obese fathers that underwent diet and/or exercise interventions may have a reduced susceptibility to develop adult chronic diseases.

It is becoming increasingly evident that at least some changes to embryo and fetal health must be resultant from the sperm health at the time of conception. Previous studies have shown that poor sperm quality including increased levels of DNA damage and/or reactive oxygen species are associated with reduced fertilisation, impaired embryonic development and increased pregnancy loss [40]–[43], [54]–[56]. Increased levels of DNA damage and ROS in sperm are commonly found in obese males [57]–[59]. Using this same model it has previously been shown that diet and exercise interventions reduced both DNA damage and ROS levels in sperm of obese male mice [13]. The improved embryo and fetal health seen in the current study may therefore be as a result of improvements to sperm DNA damage and/or ROS levels in sperm. In humans, diet and exercise interventions as well as gastric bypass surgeries have been shown to improve other sperm measures such as count, motility as well as sexual function in previously overweight and obese men [17], [18]. Further, it has been shown that the proteomic, microRNA (miRNA) and methylome content of sperm including the RNA content of testes from obese rodents are also altered [16], [60]–[64] molecular sperm signatures that have previously been shown to impact embryo health and quality [65], [66]. This may be contributing to the impaired sperm and embryo development seen in obese men. It is therefore possible that improvements to embryo and fetal health seen in the current study are resultant from additional improvements to sperm epigenetic molecular structure including changes to histone methylation and miRNA content. The specific types of molecular signatures currently altered in sperm as a result of obesity and then restored by diet and exercise interventions are currently unknown. However, a recent study in humans showed that miRNAs in serum previously altered by obesity could be restored through weight loss [67], suggesting that a similar restoration could likely occur in sperm and explain the improvements in our study.

As seen in our previous study [13] mice that underwent diet interventions (HC and HCE) had reduced levels of adiposity, improved serum cholesterol, FFAs, glucose tolerance and additionally serum leptin levels. Those receiving exercise intervention only (HE) were still obese maintaining their pre intervention level of adiposity, serum cholesterol, and impaired response to insulin, however had significant reductions in serum leptin levels which are consistent to findings found previously in obese males [68], [69]. Interventions including exercise (HE and HCE) reduced fasting serum glucose with an increased clearance of glucose after a GTT. This is likely as a result from increased glucose utilisation by skeletal muscle (reviewed by [70]). Using association analysis between the father's body composition and metabolic parameters, the data in this study suggests that the adverse embryo and fetal health due to diet induced obesity may not only be resultant from increased adiposity but additionally whole body metabolic changes. Irrespective of father's treatment or adiposity, carbohydrate and lipid profiles showed the strongest correlations to embryo and fetal health, with reduced cholesterol, and FFAs associated with improved blastocyst cell numbers and reduced cellular apoptosis. Additionally, glucose and insulin metabolism were also found to be associated with improved embryo cell numbers and fetal and placental weight ratios.

Due to lack of cytoplasmic scavenging enzymes and high levels of polyunsaturated fatty acids in their plasma membrane, sperm are highly susceptible to oxidative stress and damage [71]–[73]. Exposure to increasing levels of both cholesterol and fatty acids in human and animal sperm in vitro has previously been shown to cause increased mitochondrial ROS [74] reduced sperm motility, capacitation and fertilisation [75], [76]. In particular, cholesterol is found in the sperm membrane and its levels within the membrane affects fluidity and helps determine motility, capacitation and acrosome reaction [77], [78], all important process required for successful fertilisation [79]. Hypercholesterolemia in rabbits showed that the transport ion channels were open in the epididymis between the circulating lipids and the sperm micro environment [74] and that the sperm from these rabbits displayed reduced motility and capacitation likely caused from increasing sperm ROS. It is therefore plausible that high circulating plasma cholesterol, triglycerides and FFAs as seen in male obesity cause changes to sperm membrane dynamics and directly alter the fluidity of the plasma membrane allowing the sperm to be more susceptible ROS and DNA damage. Increased levels of ROS have been shown to change the global methylation profile of sperm [80], with hypomethylation of imprinted genes and repeat elements in sperm linked with reduced pregnancy success and increased sperm DNA damage [80]–[83]. It is therefore, possible that elevated paternal plasma glucose and lipids may in this way modifies the epigenetic profile of sperm, which is then passed onto the newly fertilised embryo, causing a delay in embryo development via alterations to paternal chromatin remodelling and indirectly reducing embryo cell numbers and implantation rates.

This is the first study to show that the impaired embryo and fetal development commonly seen in obese males can be reversed by improving their metabolic profile via exercise and diet. This study also provides the first direct evidence that the metabolic profile of obese fathers maybe a better indicator for determining the health of the resultant embryo and fetus than adiposity alone. Therefore, this study shows that exercise and diet interventions could be a combined approach to target sub fertility in overweight and obese men by improving embryo development and therefore subsequent pregnancy health. Future studies in the human are needed to determine if similar improvements can occur.

## Supporting Information

Table S1 Composition of Animal Diets.
(DOC)

Table S2 Numbers of Embryos and Pups Derived from each Father.
(DOC)

Table S3 The Effect of Diet and Exercise on Founder Male Body Composition after Intervention.
(DOC)

Table S4 The Effect of Diet and Exercise on Founder Male Serum Metabolites after Intervention.
(DOC)

Table S5 Correlations of Founder Metabolism on Blastocyst and Fetal Health Independent of Founder Adiposity.
(DOC)
