# Supplementary material for: Improving Metabolic Health in Obese Male Mice via Diet and Exercise Restores Embryo Development and Fetal Growth
Source: PLoS One. 2013 Aug 19;8(8):e71459. doi: 10.1371/journal.pone.0071459 (PMC3747240; doi:10.1371/journal.pone.0071459)
Supplement: Table S1 — Composition of Animal Diets. (DOC) [file pone.0071459.s001.doc]

Table S1: Composition of Animal Diets

| **Ingredients** | CD (SF04-057)  Control Diet | HFD (SF00-219)  Harlan Teklad TD88137 Equival | Standard Chow  (Irradiated Rat and Mouse Diet) |
| --- | --- | --- | --- |
| Sucrose (g/100g) | 34.1 | 34.1 | - |
| Casein (Acid) (g/100g) | 19.5 | 19.5 | - |
| Canola Oil (g/100g) | 6.0 | - | - |
| Clarified Butter (g/100g) | - | 21.0 | - |
| Cellulose (g/100g) | 5.0 | 5.0 | - |
| Wheat starch (g/100g) | 30.5 | 15.5 | - |
| Minerals (g/100g) | 4.9 | 4.9 | - |
| Digestible energy (MJ/kg) | 16.1 | 19.4 | 14.0 |
| Digestible energy from lipids (%) | 21.0 | 40.0 | 4.8 |
| Digestible energy form protein (%) | 14.0 | 17.0 | 20.0 |
| Digestible energy from carbohydrates (%) | 65.0 | 43.0 | 74.4 |

CD = Control diet and HFD = High fat diet. Standard Chow is the standard mouse food supplied by the University of Adelaide’s Animal House
