# Supplementary material for: Improving Metabolic Health in Obese Male Mice via Diet and Exercise Restores Embryo Development and Fetal Growth
Source: PLoS One. 2013 Aug 19;8(8):e71459. doi: 10.1371/journal.pone.0071459 (PMC3747240; doi:10.1371/journal.pone.0071459)
Supplement: Table S2 — Numbers of Embryos and Pups Derived from each Father. (DOC) [file pone.0071459.s002.doc]

Table S2: Numbers of Embryos and Pups Derived from each Father

| Father ID | Group | Number of successful matting’s | Number of embryos derived from each super ovulated mother | Number of pups derived from each father |
| --- | --- | --- | --- | --- |
| **1** | HH | 4 | 35  11  18  37 | 2 |
| **2** | HH | 2 | 26  15 | 2 |
| **3** | HH | 2 | 25  5 | 4 |
| **4** | HH | 3 | 18  36  13 | 2 |
| **5** | HH | 2 | 56  8 | 2 |
| **6** | HH | 2 | 39  16 | 1 |
| **7** | HC | 3 | 25  21  25 | 2 |
| **8** | HC | 2 | 30  8 | 1 |
| **9** | HC | 2 | 6  34 | 3 |
| **10** | HC | 3 | 6  22  27 | 3 |
| **11** | HC | 2 | 22  19 | 1 |
| **12** | HC | 2 | 38  6 | 0 |
| **13** | HC | 2 | 23  6 | 1 |
| **14** | HE | 2 | 31  36 | 2 |
| **15** | HE | 3 | 42  24  19 | 4 |
| **16** | HE | 3 | 21  5  28 | 3 |
| **17** | HE | 2 | 23  32 | 2 |
| **18** | HE | 2 | 35  26 | 0 |
| **19** | HE | 2 | 17  18 | 0 |
| **20** | HE | 2 | 37  4 | 4 |
| **21** | HCE | 2 | 27  7 | 0 |
| **22** | HCE | 4 | 23  5  21  27 | 4 |
| **23** | HCE | 2 | 18  14 | 2 |
| **24** | HCE | 3 | 37  12  18 | 1 |
| **25** | HCE | 2 | 37  5 | 3 |
| **26** | HCE | 2 | 6  37 | 1 |
| **27** | CC | 3 | 15  16  27 | 2 |
| **28** | CC | 3 | 19  4  9 | 2 |
| **29** | CC | 2 | 15  14 | 2 |
| **30** | CC | 2 | 25  7 | 1 |
| **31** | CC | 3 | 25  10  30 | 2 |
| **32** | CC | 3 | 5  29  14 | 1 |
| **33** | CC | 2 | 12  17 | 0 |
