# Supplementary material for: Improving Metabolic Health in Obese Male Mice via Diet and Exercise Restores Embryo Development and Fetal Growth
Source: PLoS One. 2013 Aug 19;8(8):e71459. doi: 10.1371/journal.pone.0071459 (PMC3747240; doi:10.1371/journal.pone.0071459)
Supplement: Table S3 — The Effect of Diet and Exercise on Founder Male Body Composition after Intervention. (DOC) [file pone.0071459.s003.doc]

***Table S3: The Effect of Diet and Exercise on Founder Male Body Composition after Intervention***

| **Diet/Intervention** | **CC** | **HH** | **HC** | **HE** | **HCE** |
| --- | --- | --- | --- | --- | --- |
| ***Pre Intervention*** |  |  |  |  |  |
| Weight (g) | 26.6 ± 0.5^a^ | 32.7 ± 0.9^b^ | 32.3 ± 1.8^b^ | 31.8 ± 1.2^b^ | 32.4 ± 1.5^b^ |
| Total Adiposity ^#^ | 14.6 ± 0.9^a^ | 25.4 ± 1.7^b^ | 24.8 ± 2.8^b^ | 24.5 ± 1.6^b^ | 25.3 ± 1.5^b^ |
| **Post Intervention** |  |  |  |  |  |
| Weight (g) | 29.9 ± 1.3^a^ | 35.1 ± 1.3^b^ | 30.2 ± 1.2^a^ | 31.6 ± 1.2^a^ | 29.3 ± 1.2^a^ |
| ***% of body weight ^#^*** |  |  |  |  |  |
| Total Adiposity | 16.7 ± 1.7^a^ | 28.0 ± 1.7^b^ | 18.1 ± 1.6^a^ | 22.3 ± 1.6^c^ | 16.8 ± 1.6^a^ |
| Lean Mass | 77.2 ± 2.0^a^ | 69.0 ± 2.0^b^ | 77.7 ± 1.8^a^ | 73.9 ± 1.8^a*^ | 79.9 ± 1.8^a^ |
| Total Bone | 1.33 ± 0.05^a^ | 1.16 ± 0.05^bc^ | 1.26 ± 0.04^ab^ | 1.13 ± 0.04^c^ | 1.32 ± 0.04^a^ |
| ***Adiposity*** |  |  |  |  |  |
| Gonadal | 2.50 ± 0.36^a^ | 4.66 ± 0.36^b^ | 2.90 ± 0.34^a^ | 3.82 ± 0.33b^ | 2.44 ± 0.33^a^ |
| ***Organs*** |  |  |  |  |  |
| Liver | 4.19 ± 0.30 | 4.09 ± 0.30 | 4.27 ± 0.29 | 3.64 ± 0.28 | 3.77 ± 0.28 |
| Pancreas | 0.48 ± 0.04 | 0.45 ± 0.04 | 0.45 ± 0.03 | 0.50 ± 0.03 | 0.47 ± 0.03 |
| Kidneys | 1.36 ± 0.06^ab^ | 1.27 ± 0.06^a^ | 1.40 ± 0.06^ab^ | 1.26 ± 0.06^a^ | 1.45 ± 0.06^b^ |
| ***Reproductive Organs*** |  |  |  |  |  |
| Testes | 0.55 ± 0.04 | 0.49 ± 0.04 | 0.55 ± 0.03 | 0.51 ± 0.03 | 0.57 ± 0.03 |
| Seminal Vesicles | 1.30 ± 0.10 | 1.13 ± 0.11 | 1.18 ± 0.10 | 1.20 ± 0.10 | 1.32 ± 0.10 |

Data is expressed as mean ± SEM per male. # measured by DEXA. Adiposity and organs data is representative of 7 CC and HCE males and 8 HH, HC and HE males. Different letters denote significance at p<0.05. ^Different to HC at p=0.07. *Different to HH at p=0.08.
