# Supplementary material for: Improving Metabolic Health in Obese Male Mice via Diet and Exercise Restores Embryo Development and Fetal Growth
Source: PLoS One. 2013 Aug 19;8(8):e71459. doi: 10.1371/journal.pone.0071459 (PMC3747240; doi:10.1371/journal.pone.0071459)
Supplement: Table S4 — The Effect of Diet and Exercise on Founder Male Serum Metabolites after Intervention. (DOC) [file pone.0071459.s004.doc]

**Table S4: The Effect of Diet and Exercise on Founder Male Serum Metabolites after Intervention**

| **Diet/Intervention** | **CC** | **HH** | **HC** | **HE** | **HCE** |
| --- | --- | --- | --- | --- | --- |
| Glucose (mmol/L^-1^) | 10.2 ± 0.6^ab^ | 10.7 ± 0.6^a^ | 9.7 ± 0.5^ab^ | 8.8 ± 0.05^b^ | 9.4 ± 0.5b^ |
| Cholesterol (mmol/L^-1^) | 3.12 ± 0.4^a^ | 4.42 ± 0.4^b^ | 3.10 ± 0.4^a^ | 4.01 ± 0.4^b*^ | 2.65 ± 0.4^a^ |
| FFA (mmol/L^-1^) | 0.92 ± 0.09 | 0.88 ± 0.09 | 0.84 ± 0.08 | 0.87 ± 0.08 | 0.81 ± 0.09 |
| Triglycerides (mmol/L-^1^) | 0.65 ± 0.05 | 0.67 ± 0.05 | 0.58 ± 0.04 | 0.68 ± 0.05 | 0.57 ± 0.04 |
| Leptin (ng/mL-1) | 3.4 ± 1.5^a^ | 16.3 ± 1.4^b^ | 5.3 ± 1.0^a^ | 5.2 ± 1.5^a^ | 3.9 ± 1.6^b^ |
| Glucose (AUC) | 1827 ± 137^ab^ | 2102 ± 138^b^ | 1680 ± 129^a^ | 1640 ± 128^a^ | 1770 ± 129a^ |
| Insulin (AAC) | 140 ± 13.8^a^ | 100 ± 13.7^bc^ | 116 ± 12.9^ab^ | 80 ± 12.8^c^ | 112 ± 12.7^abc^ |

Data is expressed as mean ± SEM per male. Serum glucose, cholesterol, FFA, triglyceride, glucose (AUC) and insulin (AAC) concentrations are representative of 7 CC and HCE males and 8 HH, HC and HE males. For serum leptin levels 4 males per treatment group were measured. Different letters denote significance at p<0.05. ^different to HH at p=0.08. *different to CC and HC at p=0.07.
