# Supplementary material for: Improving Metabolic Health in Obese Male Mice via Diet and Exercise Restores Embryo Development and Fetal Growth
Source: PLoS One. 2013 Aug 19;8(8):e71459. doi: 10.1371/journal.pone.0071459 (PMC3747240; doi:10.1371/journal.pone.0071459)
Supplement: Table S5 — Correlations of Founder Metabolism on Blastocyst and Fetal Health Independent of Founder Adiposity. (DOC) [file pone.0071459.s005.doc]

**Table S5: Correlations of Founder Metabolism on Blastocyst and Fetal Health Independent of Founder Adiposity**

| **Founder Metabolite** | **Glucose**  **(mmol/L^-1^)** | **Cholesterol**  **(mmol/L^-1^)** | **Triglycerides (mmol/L-1)*** | **FFA**  **(mmol/L^-1^)** | **Insulin**  **(AAC)^*^** |
| --- | --- | --- | --- | --- | --- |
| DNA damage cell in blastocyst |  | 0.19, p<0.01 |  |  | -0.19, p<0.01 |
| Blastocyst trophectoderm cell number |  | -0.35, p=0.02 |  |  | -0.42, p<0.01 |
| Blastocyst inner cell mass cell number |  |  | -0.24, p<0.01 |  | 0.47, p<0.01 |
| Fetal to placental weight ratio | -0.42, p<0.01 |  |  | -0.31, p<0.01 |  |
| Fetal placental weight | 0.23, p=0.04 |  |  |  |  |
| Fetal length | -0.28, p=0.05 |  |  |  |  |

Data is expressed at Pearson Correlation coefficient with corresponding p value. n=216 observations for embryo correlations and n=64 observations for fetal and placental correlations. *Correlations refer to percentage of cells within blastocyst not total cell numbers.
